# The Accuracy of Biparametric Prostate MRI in the Pathway of Patients With Prostate Cancer

**DOI:** 10.7759/cureus.83663

**Published:** 2025-05-07

**Authors:** Radu T Ion, Alexandru Serbanoiu, Alecsandra Iulia Salcianu, Maria Narcisa Popa, Florin Mihail Filipoiu, Ana M Bratu

**Affiliations:** 1 Doctoral School of Medicine, University of Medicine and Pharmacy "Carol Davila" Bucharest, Bucharest, ROU; 2 Department of Radiology, Colțea Clinical Hospital, Bucharest, ROU; 3 Department of Radiology, Emergency University Hospital Bucharest, Bucharest, ROU; 4 Department of Anatomy, University of Medicine and Pharmacy "Carol Davila" Bucharest, Bucharest, ROU; 5 Department of Radiology, University of Medicine and Pharmacy "Carol Davila" Bucharest, Bucharest, ROU

**Keywords:** biparametric mri, magnetic resonance imaging, multiparametric mri, prostate adenocarcinoma, prostate cancer

## Abstract

Background and objectives

Prostate cancer is one of the most commonly diagnosed cancers, often presenting with mild or no symptoms unless it is locally advanced or has metastasized. Given the increasing incidence of prostate cancer and its potential for severe progression, developing strategies for early detection and effective management is crucial to reduce its impact on both the population and the healthcare system. Established diagnostic methods for prostate cancer include prostate-specific antigen testing, digital rectal examination, ultrasound-guided biopsy, and histological analysis. MRI plays a key role in localizing prostate cancer, assessing its extent, and predicting tumor aggressiveness. Both multiparametric MRI and biparametric MRI (bpMRI) are currently in use. This study aims to evaluate the role and accuracy of bpMRI in patients with prostate cancer, particularly for diagnosing clinically significant cancer, assessing its extent, and monitoring patients post-treatment for early recurrence.

Materials and methods

This retrospective study analyzed data from 50 patients who underwent MRI examinations. The prostate lesions identified in these patients were later pathologically confirmed as prostate cancer through biopsy or surgical resection. The study aimed to evaluate the accuracy of T2-weighted imaging (T2WI), diffusion-weighted imaging, apparent diffusion coefficient (ADC) maps, and T1-weighted imaging (T1WI).

Results

On T2WI, the lesions appeared hypointense in 46 (92%) of the 50 cases. Diffusion restriction was observed on DWI and ADC map sequences in 43 (86%) of the cases. On T1WI, cancerous lesions were isointense in 47 (94%) cases and moderately hyperintense in three (6%) cases, particularly in lesions larger than 8 mm in diameter.

Conclusions

The findings of this study confirm the high accuracy of T2WI in diagnosing malignant prostate lesions. The MRI information obtained is crucial for treatment planning and for assessing patient prognosis.

## Introduction

Prostate cancer is the fourth most common type of malignancy worldwide, following lung, breast, and colorectal cancers, according to the International Agency for Research on Cancer via the Global Cancer Observatory in 2022. Among men, it ranks as the second most frequently diagnosed cancer after colorectal cancer. Globally, prostate cancer is the eighth leading cause of cancer-related death across both sexes and all age groups - following lung, colorectal, liver, breast, stomach, pancreatic, and esophageal cancers - and the fifth leading cause of cancer death in men, after lung, liver, colorectal, and stomach cancers [1].

According to the American Cancer Society’s Cancer Statistics Center, prostate cancer is projected to be the second most commonly diagnosed malignancy in 2024, following breast cancer. It is also expected to be the fifth leading cause of cancer-related death overall, after lung and bronchus, colorectal, pancreatic, and breast cancers, and the second leading cause of cancer death in men, after lung and bronchus cancer [2].

Several factors are associated with an increased risk of developing prostate cancer, including age, race (particularly among men of African descent), and having a first-degree relative with the disease [3]. Additional risk factors include obesity, BRCA2 gene mutations, and less common conditions such as Lynch syndrome [3].

Given the rising incidence of prostate cancer and its potential for severe progression, developing strategies for early detection and effective management is essential to reduce its impact on both the population and healthcare systems. One challenge in diagnosing prostate cancer is that patients are often asymptomatic or present with nonspecific lower urinary tract symptoms, unless the cancer is locally advanced or has metastasized [3]. Established diagnostic methods for prostate cancer include prostate-specific antigen (PSA) testing, digital rectal examination (DRE), and ultrasound-guided biopsy with subsequent histological examination [4,5]. Recently, MRI has become increasingly important for localizing prostate cancer, assessing its extent, and predicting tumor aggressiveness [5].

The suspicion of prostate cancer often arises from elevated serum PSA levels, although this biomarker has low specificity and positive predictive value [3,4,6,7]. There is ongoing debate about whether PSA screening reduces mortality, but many experts agree that it can be used for screening in men at average risk, typically between the ages of 55 and 69 [4,8]. To improve sensitivity and specificity, PSA density and velocity can be evaluated, and different PSA cutoff values can be applied based on age [3,9]. Various PSA cutoff levels are used to recommend prostate biopsy [3].

On DRE, features that raise suspicion for prostate cancer include a palpable nodule or a hard, asymmetrical, or enlarged gland. In some cases, the prostate may appear normal on DRE [3]. After suspicion arises from elevated PSA and DRE findings, biopsy with histopathologic evaluation is generally performed. Histological examination reveals that most prostate cancers are multifocal, with 75-80% occurring in the peripheral zone, typically in a posterior or posterolateral location. Clinically significant prostate cancers are often detected in this area. Prostate cancer may also develop in the transition zone, though these cases tend to have more favorable pathological characteristics and better survival rates without recurrence [3]. Transrectal biopsy, guided by ultrasound with a 10- to 12-core technique, is commonly used, with targeted biopsies taken from hypoechoic regions. However, this method has limitations in sensitivity and specificity. To reduce the risk of infection and bleeding, some institutions use transperineal needle biopsy [3,9,10]. In addition to ultrasound-guided biopsies, MRI is increasingly used to detect suspicious areas in the prostate and guide biopsies to these specific regions [3,9,10]. MRI is the most accurate imaging tool for both anatomical assessment and detecting potential malignancies in the prostate [9].

After biopsy, the Gleason score and International Society of Urological Pathology (ISUP) grade are used to assess prostate cancer based on tumor architecture [2,11,12]. Not all patients with suspected prostate cancer follow the typical diagnostic pathway of biopsy after elevated PSA, as an elevated PSA does not necessarily indicate prostate cancer. In cases where MRI does not show any suspicious regions, unnecessary biopsies and their associated risks can be avoided [6]. Additionally, some men are diagnosed with prostate cancer incidentally following transurethral resections [3].

Thanks to PSA screening and improved therapeutic methods, prostate cancer mortality has declined [4]. However, the number of patients diagnosed and treated for indolent or low-volume tumors has increased [4,13]. Given the significant morbidity associated with treatment, careful consideration is necessary when deciding whether patients should undergo prostatectomy or if active surveillance or focal therapy might be more appropriate, particularly for the low- to intermediate-risk group [10]. Gleason grade is used to stratify patients based on histopathological examination. Tumors with a Gleason score ≤6 are considered well differentiated (low grade), those with a score of 7 are classified as moderately differentiated (intermediate grade), and tumors with a score ≥8 to 10 are considered poorly differentiated (high grade) [9]. Tumors with a Gleason score of 7 or higher are classified as clinically significant [9]. Other criteria for defining clinically significant prostate cancer include a histopathological ISUP grade ≥ 2, tumor volume ≥ 0.5 cc, and/or extra-prostatic extension [1].

## Materials and methods

A retrospective review of the oncologic database at our institution, spanning from January 2016 to November 2023, included 50 men aged 50-84 years who underwent MRI examinations using a 1.5 T magnetic field strength scanner (Siemens Magnetom Lumina, Siemens Healthineers, Erlangen, Germany). To protect patient privacy and ensure their identities remained confidential, all medical records were de-identified. MRI sequences included T1-weighted imaging (T1WI), T2-weighted imaging (T2WI), and diffusion-weighted imaging (DWI), along with apparent diffusion coefficient (ADC) maps in both axial and coronal planes. The field of view was approximately 180-200 mm, centered on the prostate, with a larger field of view around 350-450 mm to assess the bony pelvis for metastases.

Prostate cancer in suspicious MRI lesions was confirmed through histopathological examination of specimens obtained via radical prostatectomy, ultrasound-guided prostate biopsy, and, to a lesser extent, tissue samples from transurethral resection of the prostate (TURP). In most cases, confirmation was based on radical prostatectomy specimens.

Prostate biopsy was performed in three categories of patients who were not candidates for radical prostatectomy. The first category included patients with locally advanced disease in whom achieving negative surgical margins was not feasible. The second category involved patients with lymph node and distant metastases, where biopsy was performed to confirm the prostatic origin of the tumor. The third category consisted of patients with significant comorbidities for whom radical prostatectomy posed a high risk. TURP tissue samples were used in a minority of cases, particularly in patients with advanced prostate cancer in the central gland, where TURP was employed as a palliative measure to alleviate urinary symptoms. No patients were excluded from the study.

Clinical, pathological, and imaging parameters were collected from electronic medical records. All records used in this study have been de-identified, and the data were compiled into a Microsoft Excel spreadsheet (Microsoft Corporation, Redmond, WA, USA).

## Results

As shown in Table 1, T2WI revealed hypointense lesions in 46 (92%) of the 50 cases. Diffusion restriction on DWI and ADC map sequences was observed in 43 (86%) of the 50 cases. On T1WI, cancerous lesions appeared isointense in 47 (94%) of the cases and moderately hyperintense in three (6%) cases with lesions larger than 8 mm in diameter. On T2WI, prostate carcinoma presented as hypointense in 46 (92%) of the 50 cases, isointense in one patient (0.5%), and hyperintense in three patients (6%). Diffusion restriction on DWI and ADC map sequences was observed in 43 (86%) of the 50 cases, showing slightly lower accuracy compared to T2WI. As previously mentioned, T1WI was less reliable in assessing cancerous lesions, with the majority appearing isointense in 47 (94%) of the cases and moderately hyperintense in three (6%) cases with lesions larger than 8 mm.

**Table 1:** MRI aspect on T1WI, T2WI, DWI, and ADC map ADC, apparent diffusion coefficient; DWI, diffusion-weighted imaging; T1WI, T1-weighted imaging; T2WI, T2-weighted imaging

MRI sequence	Hypointense	Isointense	Hyperintense
T1WI	0 (0%)	48 (96%)	2 (4%)
T2WI	46 (92%)	1 (2%)	3 (6%)
DWI	2 (4%)	5 (10%)	43 (86%)
ADC	45 (90%)	5 (10%)	0 (0%)

**Figure 1:**
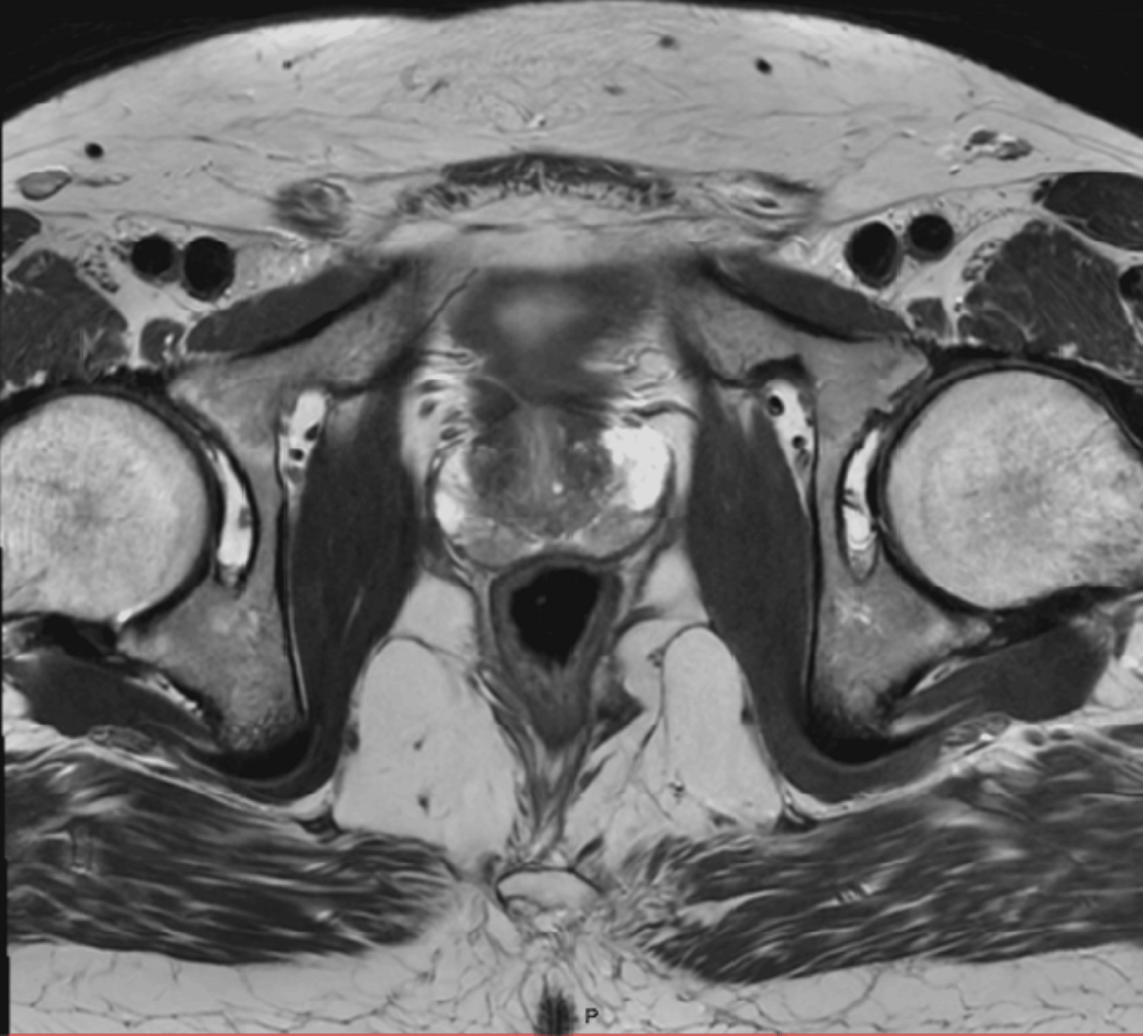
Characteristic appearance of the “organized chaos” in BPH seen on T2WI BPH, benign prostatic hyperplasia; T2WI, T2-weighted imaging

**Figure 2:**
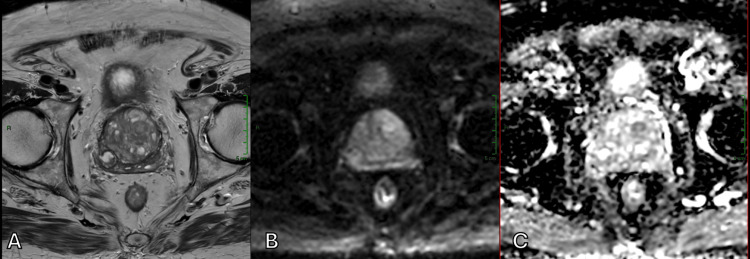
Appearance of “disrupted organized chaos” by a hypointense lesion on T2WI, which shows restricted diffusion with high signal on DWI and reduced values on the ADC map, located in the left transition zone (A) Axial T2WI. (B) Axial DWI. (C) Axial ADC map. ADC, apparent diffusion coefficient; DWI, diffusion-weighted imaging; T2WI, T2-weighted imaging

Most patients in our study presented with benign prostatic hyperplasia (BPH), as shown in Figure 1. This allowed us to evaluate and compare the “organized chaos” observed in the transition zone in BPH cases with the “disruption of organized chaos” characteristic of transition zone involvement in prostate cancer, as demonstrated in Figure 2. 

**Figure 3:**
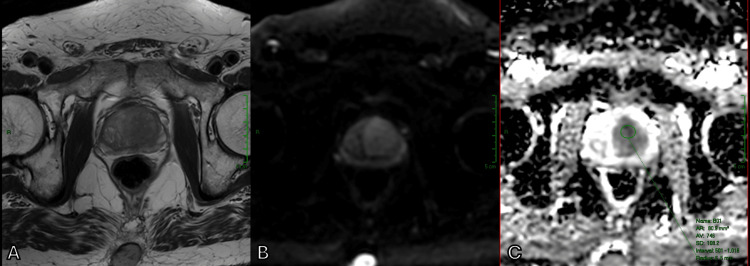
T2WI (A) in correlation with DWI (B) showing a large left transition zone prostate cancer with ADC (C) values of 746 × 10⁻⁶ mm²/s (A) Axial T2WI. (B) Axial DWI. (C) Axial ADC map. ADC, apparent diffusion coefficient; DWI, diffusion-weighted imaging; T2WI, T2-weighted imaging

**Figure 4:**
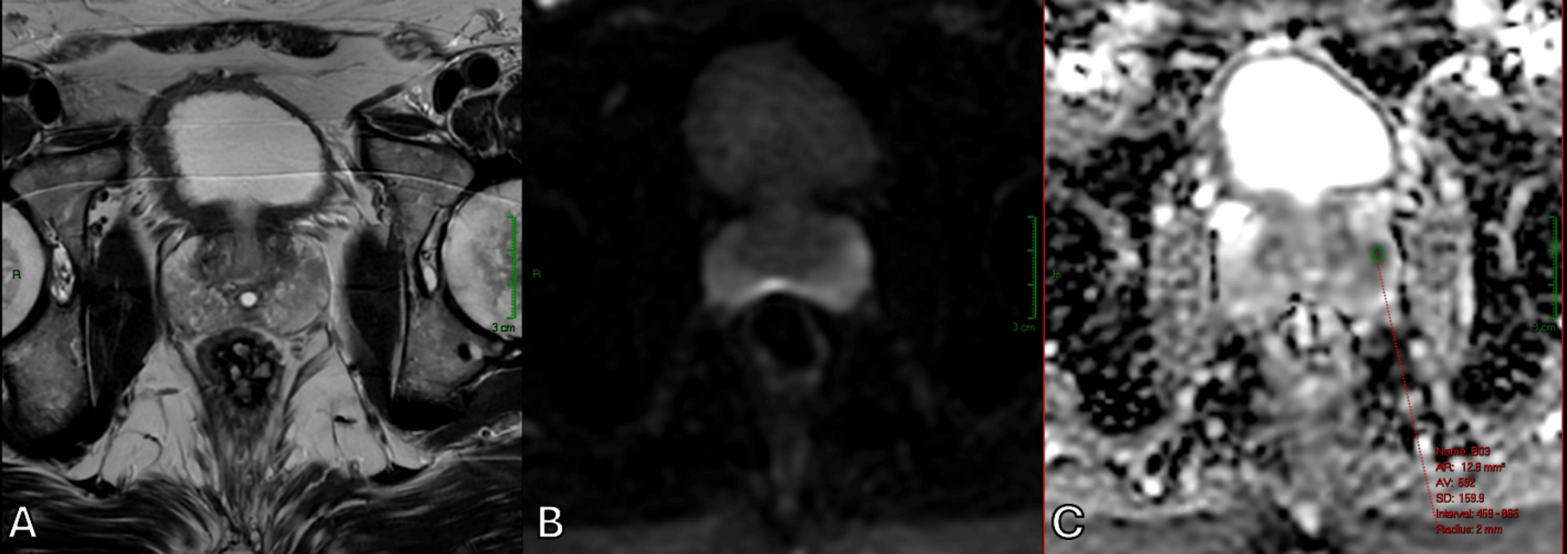
T2WI in correlation with DWI, showing a left peripheral zone prostate cancer with ADC values of 592 × 10⁻⁶ mm²/s (A) Axial T2WI. (B) Axial DWI. (C) Axial ADC map. ADC, apparent diffusion coefficient; DWI, diffusion-weighted imaging; T2WI, T2-weighted imaging

In addition to identifying a suspicious nodule within the transition zone affected by BPH, DWI and ADC maps were also helpful in assessing the aggressiveness of the lesion by measuring ADC values. While both Figure 3 and Figure 4 illustrate malignant prostate tumors, Figure 4 shows a much lower ADC value, which corresponds to a higher Gleason score, indicating a higher likelihood of clinically significant cancer.

**Figure 5:**
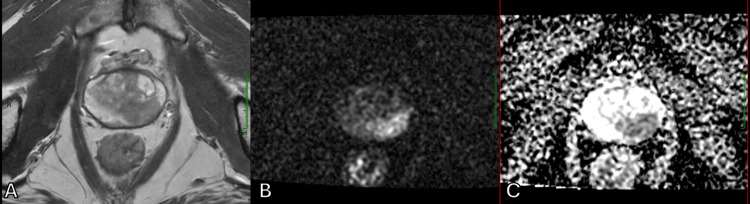
T2WI and DWI sequences, along with the ADC map, demonstrate a lentiform nodule in the left peripheral zone showing restricted diffusion and measuring 1.9/1.0 cm in the axial plane (A) Axial T2WI. (B) Axial DWI. (C) Axial ADC map. ADC, apparent diffusion coefficient; DWI, diffusion-weighted imaging; T2WI, T2-weighted imaging

To assess the risk of significant prostate cancer in our patients, we applied the Prostate Imaging Reporting and Data System (PI-RADS) v2.1 criteria. In Figure 5, a suspicious nodule is seen in the left part of the peripheral zone, demonstrating a lenticular shape and hypointensity on T2WI. The lesion showed reduced ADC values, indicating restricted diffusion, and measured 1.9/1.0 cm in the axial plane, which is greater than 1.5 cm, thus falling into the PI-RADS 5 category, corresponding to a very high likelihood of clinically significant cancer.

**Figure 6:**
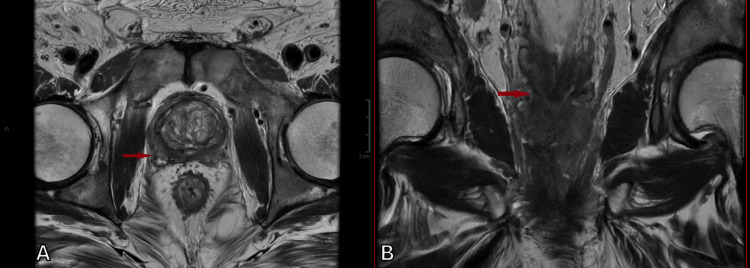
Extraprostatic involvement from prostate cancer (indicated by the red arrow) arising from the right peripheral zone, with bulging of the prostatic capsule and superior extension into and invasion of the right seminal vesicle, as seen in axial and coronal T2WI (A) Axial T2WI. (B) Coronal T2WI. T2WI, T2-weighted imaging

After evaluating the prostate gland and the characteristics of nodules with a high probability of malignancy, the next step was to assess the potential extension to adjacent structures. Due to their excellent anatomical detail, T2WI sequences in both axial and coronal planes are highly suitable for this purpose. In Figure 6, extraprostatic extension is suggested by the bulging of the right contour of the gland and the invasion of the right seminal vesicle.

**Figure 7:**
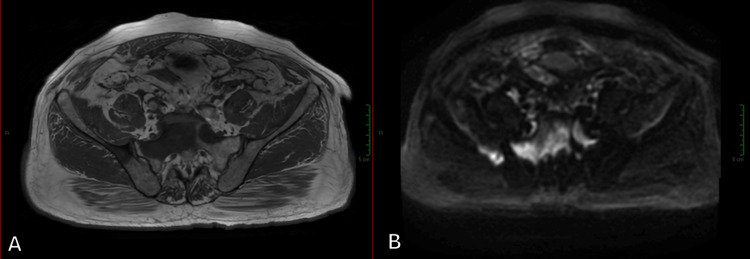
Bone metastases affecting the sacrum and right iliac body and wing, appearing as hypointense areas on T1WI with associated restricted diffusion on DWI (A) Axial TWI. (B) Axial DWI. DWI, diffusion-weighted imaging; T1WI, T1-weighted imaging

Given prostate cancer’s known propensity for metastasizing to bone, we assessed the bony pelvis in all patients with suspected prostate cancer to rule out or further characterize any suspicious lesions, as demonstrated in Figure 7.

**Figure 8:**
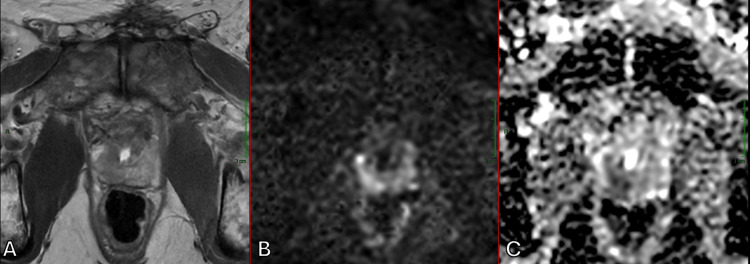
Small areas of tumor recurrence after radical prostatectomy, seen as low signal areas on T2WI with restricted diffusion, illustrated as high signal intensity on DWI and low ADC values, located posteromedial and posterolateral to the prostatic urethra (A) Axial T2WI. (B) Axial DWI. (C) Axial ADC map. ADC, apparent diffusion coefficient; DWI, diffusion-weighted imaging; T2WI, T2-weighted imaging

Additionally, our study included post-therapeutic monitoring to ensure the early detection of local recurrence after radical prostatectomy, as shown in Figure 8.

## Discussion

MRI protocol

In the current study, we aimed to evaluate the accuracy of biparametric MRI (bpMRI) in diagnosing prostate cancer, comparing our results with those in the specialized literature. Proper characterization of suspicious prostate lesions on MRI requires knowledge of prostatic anatomy and pathology, the MRI appearance of prostate cancer, and management strategies post-diagnosis to differentiate between normal and abnormal post-treatment appearances. Our study evaluated the MRI features of prostatic adenocarcinoma in 50 patients using T2WI, DWI, and ADC maps. We found that T2WI demonstrated high accuracy, raising the suspicion of prostate cancer in all cases.

While T1-weighted images have limited utility in evaluating the prostate gland, they can effectively identify hemorrhage after biopsy. Hemorrhage, which may extend beyond the needle track due to prostatic cells producing citrate (an anticoagulant), can obscure tumors. It is recommended to wait six to eight weeks before performing an MRI, as hemorrhage persists only in a minority of cases. T1WI is also useful for assessing regional lymph node and bone metastases [4,13-15].

T2WI provides both anatomical and morphological details of the prostate gland and its relationship with surrounding structures [15]. The normal peripheral zone appears homogeneously hyperintense on T2WI due to its dense glandular and ductal tissue and high water content [4,16]. The transition zone has a heterogeneous signal intensity due to epithelial and stromal hyperplasia in benign prostatic hypertrophy, creating an “organized chaos” pattern [4,16]. The central zone, with its fibrous tissue content, appears hypointense on T2WI, as does the pseudo-capsule surrounding the gland [16]. Histological features of prostate cancer are mirrored in its MRI appearance. The densely packed malignant cells and reduced water content result in hypointensity on MRI, with lower intensity corresponding to higher tumor aggressiveness [4]. In the transition zone, certain features should raise suspicion for prostate cancer on T2WI, even in the absence of DWI or ADC abnormalities. These include “disruption of organized chaos,” obscured margins, a lenticular or fusiform shape, and focal homogeneous intermediate to low signal, also known as the “erased charcoal drawing sign” [9,16,17].

Not every abnormality detected on T2WI is indicative of prostate cancer. Other conditions that may mimic malignancy include acute, chronic, or granulomatous prostatitis; post-biopsy hemorrhage; radiation effects; scars; atrophy; and hormonal therapy [4,16]. Prostatitis may appear hypointense on T2WI and low on the ADC map, with contrast enhancement on dynamic contrast-enhanced imaging (DCEI). It typically presents as a band or wedge shape or involves the prostate diffusely, in contrast to prostate cancer, which tends to present as a round, oval, or irregular lesion with significantly lower ADC [9,16]. Along with assessing the prostate gland and suspicious nodules, it is also essential to evaluate the length of the membranous urethra, as this is a predictor of continence three to six months post-prostatectomy [10].

DWI shows water molecule motion within both malignant and nonmalignant prostatic tissue. Prostate cancer is characterized by high cellularity within the glandular epithelium, restricting water molecule movement [4,16]. DWI, when combined with various magnetic gradient strengths and b-values, produces the ADC map, which is low in areas with restricted water movement, such as tumors [4]. Hyperintensity on DWI with low ADC values suggests a malignant process. It has been shown that lower ADC values correlate with higher Gleason scores, making DWI a crucial tool for assessing tumor aggressiveness [4,16]. However, DWI has limitations, such as potential restricted diffusion in BPH and susceptibility to artifacts, including bowel peristalsis, gas in the rectum, or the presence of hip prostheses [16]. In our study, the DWI/ADC map alone showed high accuracy for detecting malignant lesions, though it was less accurate than T2WI. When DWI/ADC maps were combined with T2WI, the diagnostic accuracy for prostate cancer was highest. In agreement with Pesapane et al., no high-grade cancers were missed in our study when using bpMRI [6]. High-grade tumors, including those with a Gleason score of 8 or higher and extraprostatic extension, were identified using DWI/ADC maps alongside T2WI.

As Palumbo et al. reported, the combination of T2WI and DWI with the ADC map is sufficient for detecting prostate cancer and assessing lesion aggressiveness [17]. Our results align with this, as ADC values correlated well with histopathological findings.

DCEI, which involves T1-weighted images obtained after intravenous gadolinium-based contrast agents, can reveal intense and early enhancement, strongly suggesting prostate cancer. However, prostatitis and hyperplastic nodules may also show this pattern [4]. Despite its utility, DCEI changes the MRI diagnosis in only a minority of cases. Recent guidelines have reduced its role in prostate cancer imaging, recommending its use only when T2WI and DWI sequences are of inadequate quality or when uncertainty remains after evaluating these sequences in suspected peripheral zone tumors. Its role in evaluating the transition zone is limited [4,5,6,9]. Although prostate cancer originating in the central zone is rare, it may involve the central zone when tumors extend from the peripheral or transition zones. In such cases, even if T2WI, DWI, and ADC maps show no abnormalities, DCEI may reveal early focal enhancement [9]. DCEI is also useful in detecting local recurrence in patients who have undergone radical prostatectomy, focal therapy, or transurethral resection, as these treatments alter the zonal anatomy [4,9,16]. After external beam radiation therapy, both DCEI and DWI are effective in detecting recurrence, as they provide more detailed information than T2WI [10]. DCEI is also helpful after brachytherapy, where radioactive seeds cause artifacts in DWI [10].

Magnetic resonance spectroscopy imaging (MRSI) can assess citrate and choline levels, which may indicate prostate cancer in areas with abnormal metabolite expression. While MRSI has a limited role in routine prostate cancer evaluation, it is still used in research [4].

Currently, multiparametric MRI includes T1 and T2WI, DWI, and DCEI, while bpMRI includes only T2WI and DWI [6,13,18,19]. Some studies suggest that detecting clinically significant prostate cancer with bpMRI is comparable to multiparametric MRI, and we prefer using the latter in daily practice for patients with elevated PSA [6,18,20]. The advantages of bpMRI include similar detection rates but with fewer false positives, lower cost, reduced acquisition time (less than 15 minutes), and the absence of risks associated with gadolinium-based contrast agents, such as intravenous catheterization, nephrogenic systemic fibrosis, gadolinium retention, and deposition in the brain and other tissues [5,6,19,21]. Gadolinium-based contrast agents pose risks even in patients with normal renal function when used repeatedly [6]. However, DCEI remains important, particularly when DWI cannot be assessed due to poor signal-to-noise ratios or artifacts [9,19], and is crucial in detecting local recurrence in treated patients [19].

To improve MRI use in managing prostate cancer, the PI-RADS offers guidelines on the role of each sequence and its appropriate use, with the latest version being PI-RADS v2.1 [9]. Song et al. found that the diagnostic performance of bpMRI using the PI-RADS v2.1 criteria was lower than that of mpMRI, but still sufficient to make bpMRI a viable screening tool for clinically significant prostate cancer [21]. In our study, applying the PI-RADS v2.1 system on bpMRI helped determine the likelihood of clinically significant prostate malignancies and identify all clinically significant cancers.

The role of MRI in assessing prostate cancer extension, local, and distant metastases

Once prostate cancer is suspected based on MRI findings, the next step is to assess the extent of the tumor. MRI can suggest extra-prostatic involvement through several key features, such as the loss of the rectoprostatic angle, irregular margins, bulging of the gland’s contour, asymmetry of the neurovascular bundles, and involvement of the bundles themselves [3]. However, asymmetry of the prostate gland alone should not be considered suspicious, as it is a common finding in BPH [9].

In the evaluation of the seminal vesicles, invasion is indicated by an abnormally low T2-weighted signal, restricted diffusion, or contrast enhancement in this region [4]. Features such as extraprostatic extension, neurovascular bundle and seminal vesicle invasion, larger tumor size, lymphatic metastases, and positive surgical margins all point to locally advanced disease and are associated with a higher likelihood of local recurrence [22]. Additionally, the anatomical absence of the prostatic pseudocapsule at the apex is also associated with an increased risk of local recurrence [22].

For distant metastasis evaluation, particularly to the bone, studies suggest that whole-body MRI with DWI, along with scintigraphy CT and PET/CT, can be valuable tools [3]. Some studies also support the inclusion of DCEI when evaluating bone and lymph node metastases, as it may provide additional useful information and should be incorporated into the imaging protocol [3,4].

Treatment of prostate cancer and post-treatment evaluation

The treatment approach for prostate cancer is personalized based on the cancer’s extent and preoperative risk stratification. Available treatment options include active surveillance, focal therapies (such as cryotherapy and high-intensity ultrasound), radical prostatectomy, radiotherapy (both brachytherapy and external beam radiotherapy), hormone ablation therapy (including antiandrogens and luteinizing hormone-releasing hormone analogues), and chemotherapy. For tumors localized to the prostate, the preferred treatments are radical prostatectomy and radiotherapy, often combined with hormone therapy [3,22].

In the case of recurrent disease, certain surgical procedures, particularly those preserving the neurovascular bundles and urethral sphincter, may be associated with local recurrence after therapy. Pelvic recurrence can also manifest through metastases to lymph nodes and bones. This highlights the importance of monitoring patients with treated prostate cancer through regular check-ups to detect recurrence early. In many cases, due to the small size of relapsed tumor tissue, DRE or imaging may not be able to detect the disease, making biopsy guidance challenging. Early detection of recurrence enables the use of surgery, radiotherapy, or cryoablation for local recurrence, while systemic therapy is utilized for distant metastases [22].

Limitations

A limitation of our study is that all patients examined were referred to our institution for imaging studies due to a high suspicion of prostate cancer based on clinical (symptoms and DRE) and biochemical (PSA levels) factors. Therefore, the collaboration with an experienced clinician who can accurately identify patients with a high likelihood of prostate cancer might influence the MRI diagnosis. There is a possibility that the radiologist could be biased toward classifying suspicious and indeterminate lesions as cancer, even when the MRI findings may be more consistent with other diagnoses, such as acute or chronic prostatitis. To assess the overall effectiveness of bpMRI, a larger, more diverse cohort of men should be evaluated, including those with prostate cancer, those with non-cancerous prostate pathology, and those with no apparent clinical or biochemical abnormalities. Moreover, the radiologist should be blinded to the clinical suspicion and the proportion of patients with prostate malignancy to avoid bias and ensure both benign and malignant diagnoses are considered for indeterminate MRI lesions.

## Conclusions

This study aligns with existing literature regarding bpMRI as an accurate and effective method for evaluating patients with suspected prostate cancer. bpMRI can be used in patients with a low likelihood of prostate malignancy to avoid unnecessary biopsies, and in patients with a high likelihood of cancer, to properly assess the extent of the disease and guide appropriate treatment.

Furthermore, compared to multiparametric MRI, bpMRI offers reduced costs, higher patient acceptance, and a lower impact on the medical system. It also has a shorter examination time, better patient compliance, fewer motion artifacts, and produces high-quality images. The absence of gadolinium-based contrast agents eliminates the associated risks, especially in patients with kidney disease or those requiring frequent MRIs with normal renal function.
